# A conformal multi-port MIMO patch antenna for 5G wireless devices

**DOI:** 10.1371/journal.pone.0295358

**Published:** 2023-12-07

**Authors:** Tu Chu-Anh, Phuong Kim-Thi, Hung Nguyen-Manh, Hung Tran-Huy

**Affiliations:** 1 Faculty of Electrical and Electronic Engineering, PHENIKAA University, Hanoi, Vietnam; 2 Faculty of Electrical and Electronics Engineering, ThuyLoi University, Hanoi, Vietnam; 3 Faculty of Electrical and Electronics Engineering, Hanoi Open University, Hanoi, Vietnam; Chitkara University, INDIA

## Abstract

This paper presents a multiple-input-multiple-output (MIMO) antenna array with low-profile and flexible characteristics. Multiple microstrip patches are arranged in the E-plane configuration and decoupled by shorted quarter-wavelength stubs. The antenna has a small element spacing of 0.032 λ, where λ is a free-space wavelength at the center frequency. To demonstrate the feasibility of the proposed concept, a 1 × 4 MIMO array prototype is fabricated. The measured results on the fabricated prototype demonstrate that the MIMO antenna has good operation features at 4.8 GHz with a reflection coefficient of less than –10 dB and an isolation of better than 20 dB. Besides, good radiation patterns and broadside gain of around 4.5 dBi are also attained. The antenna also works in the bending mode and has the capability of extending to large-scale MIMO arrays. Such attractive features prove the utility of the proposed antenna in various modern electronic devices.

## Introduction

Multiple-input-multiple-output (MIMO) antenna has achieved extensive research as an effective solution for 5G wireless communication services since it can increase the channel capacity without additional spectrum and alleviate the multipath fading problem [1, 2]. Among many types of antenna structures, the microstrip patch is extensively utilized due to its flat, low-profile design, making it easily integrated with a wide range of electronic devices [3]. When multiple antennas are positioned close to each other, high mutual coupling will significantly deteriorate the entire system’s performance. Consequently, there is significant ongoing research in reducing the mutual coupling between MIMO elements [4].

There are various decoupling structures reported in the literature for mutual coupling reduction. They can be roughly divided into four categories. The first type is the counteraction scheme, which creates additional coupling paths between the MIMO elements to counteract the original coupling paths. This can be achieved with the aid of neutralization lines [5, 6], parasitic elements [7], or dielectric superstrate [8, 9]. The second decoupling technique is to directly suppress the original coupling between the MIMO elements. The authors proposed defected ground structures in [10, 11], metamaterials [12–14]. Another decoupling type is to excite orthogonal operating modes on the MIMO elements. Near-field resonators [15] and interdigital lines [16] are effective decoupling structures. Alternatively, the fourth decoupling scheme, namely the self-decoupling method, is utilized in [17–20]. In such designs, the intrinsic characteristics of the antenna itself are fully utilized to improve the antenna isolation, rather than using external decoupling networks. To conclude, the first three decoupling schemes have the advantages of high isolation improvement, but complexity and/or high-profile decoupling structures are the critical drawbacks. On the other hand, the self-decoupling technique does not require any decoupling structure, leading to a decrease in the design complexity. However, this method suffers from a large element spacing and low isolation improvement.

It is worth noting that although high isolation can be achieved, most of the above-mentioned MIMO antennas are not suitable for flexible wireless systems, which are rapidly increasing. Nowadays, flexible antennas are in great demand for on-body and off-body communication, wearable sensing systems, radio frequency identification (RFID), and wireless body area networks (WBAN) [21, 22]. Therefore, they are important for the development of next-generation technologies for the improvement of human life. Various flexible MIMO antennas using monopole structures have been reported in the open literature [23–25]. However, the integration of a monopole antenna in electronic devices is more difficult than the microstrip patch antenna. This is due to the fact that the performance of the monopole antenna is significantly affected when mounting on a metallic surface. To the best of the authors’ knowledge, only several MIMO patch antennas with flexible characteristics have been published [26–28]. Nonetheless, high profile and large element spacing are drawbacks of the designs [26, 27]. Besides, the capability of extending to multiple ports MIMO is not investigated in such designs.

In this paper, a low-profile and flexible MIMO antenna is presented for operation at the N79-5G frequency band. The MIMO array consists of multiple microstrip patch elements arranged in the E-plane configuration. For mutual coupling reduction, the shorted quarter-wavelength stubs are inserted between the radiating patches. This contributes to producing a perpendicular mode on the non-exited element, rather than a similar operating mode as the excited element. The antenna is first designed and investigated with a two-element array. Then, further increment in the number of MIMO elements is considered. Finally, a four-element MIMO antenna array is fabricated and tested to validate the design concept. The measurements demonstrate the capability of working in both normal and bending modes of the proposed antenna. In comparison with the reported decoupling networks for microstrip patch antenna, the proposed decoupling structure has several advantages including high isolation improvement with small element spacing, applicable for multi-element MIMO array as well as its flexible capability.

## Two-element E-plane MIMO array

### Antenna design

**Fig 1:**
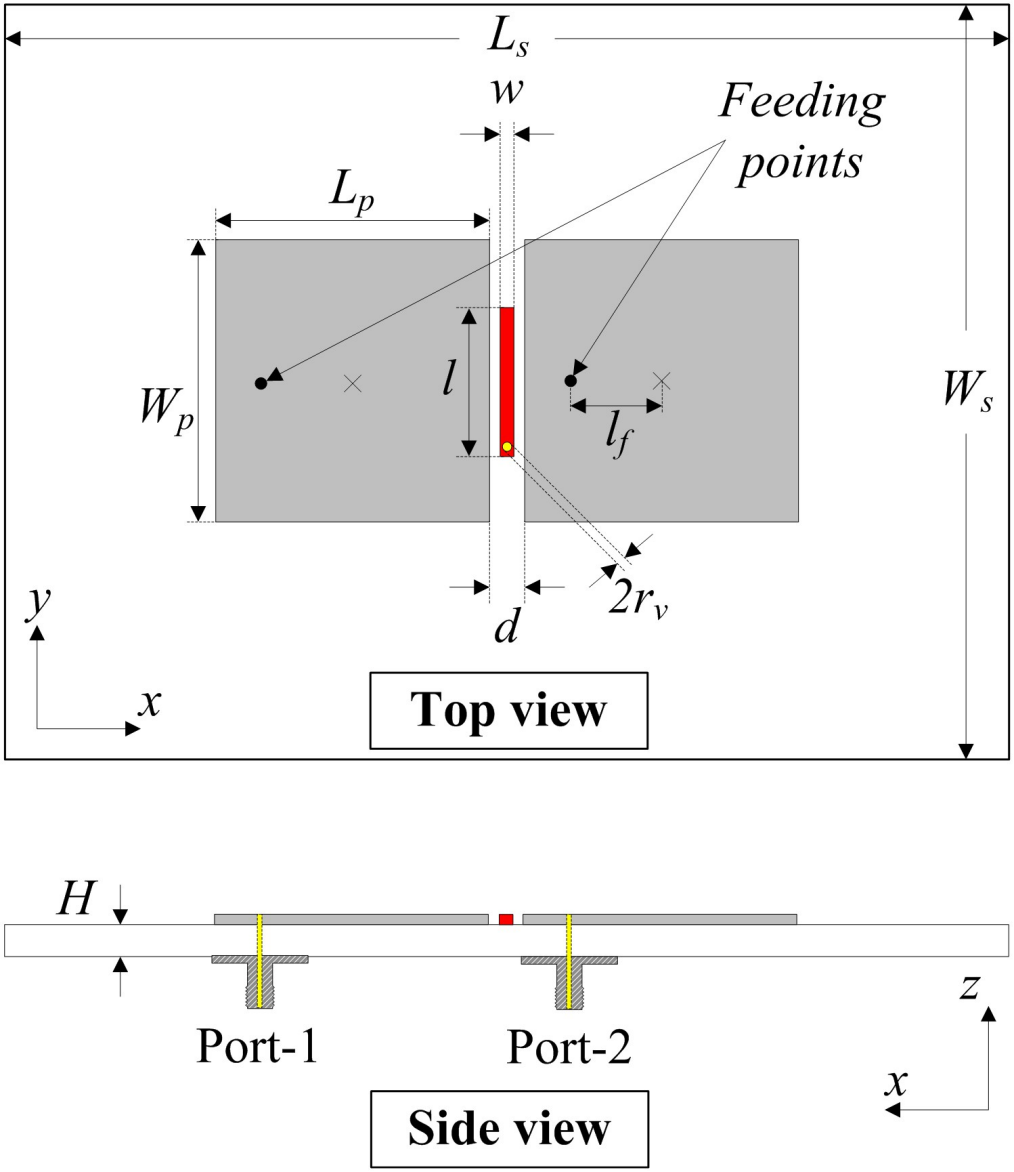
Geometry of the proposed 2-element MIMO array.

Fig 1 presents the configuration in terms of top and cross-section views of the proposed two-element MIMO array. The microstrip patches are arranged in the E-plane configuration. The patch’s length is chosen about half-effective wavelength at the desired frequency, 4.8 GHz. Two 50-*Ω* SMA connectors are connected to the patches. To suppress the mutual coupling, a quarter-wavelength shorted stub is located between the MIMO elements. The antenna is designed on the ROGER-5880 substrate with a dielectric constant of 2.2. The optimized dimensions are as follows: *L*_*s*_ = 80, *W*_*s*_ = 60, *H* = 0.5, *L*_*p*_ = 20.3, *W*_*p*_ = 20.6, *l*_*f*_ = 3.6, *d* = 2.0, *r*_*v*_ = 0.2, *l* = 12.0, *w* = 0.8 (unit: mm).

### Decoupling mechanism

Assume that there are two antennas working in transmitting and receiving modes [29]. If the polarizations are matched between the transmitting and receiving antennas, the receiving antenna will extract maximum power from the incoming waves. In contrast, when the polarizations are in a perpendicular arrangement, no power is received.

**Fig 2:**
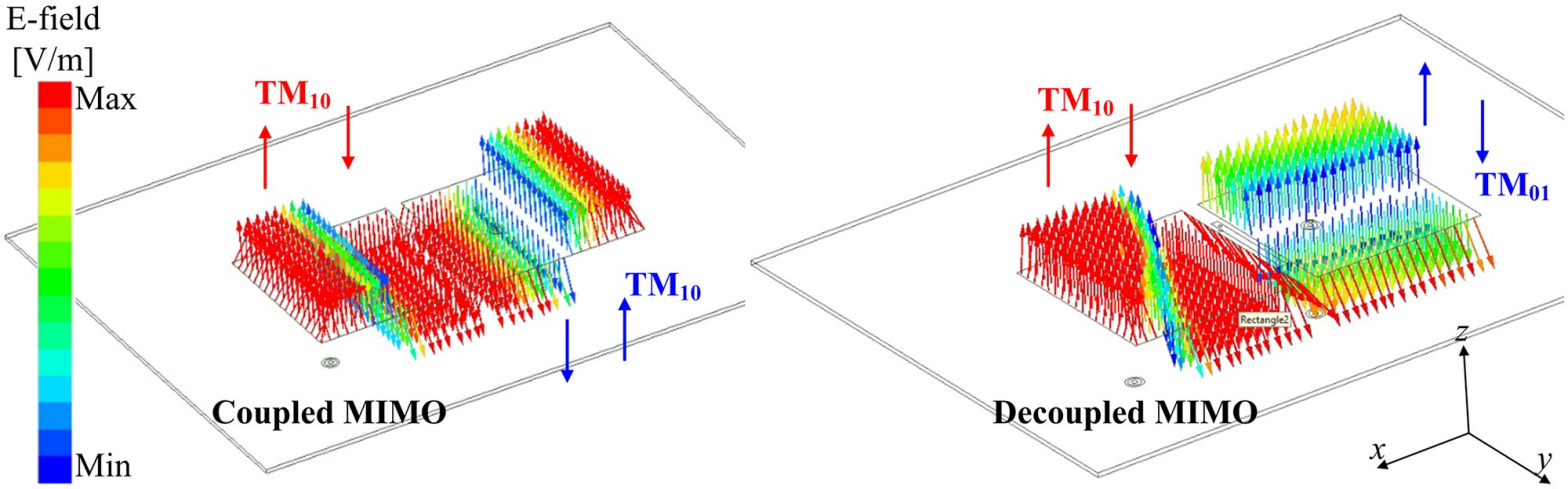
Simulate E-field distribution at 4.8 GHz of coupled and decoupled MIMO antennas.

**Fig 3:**
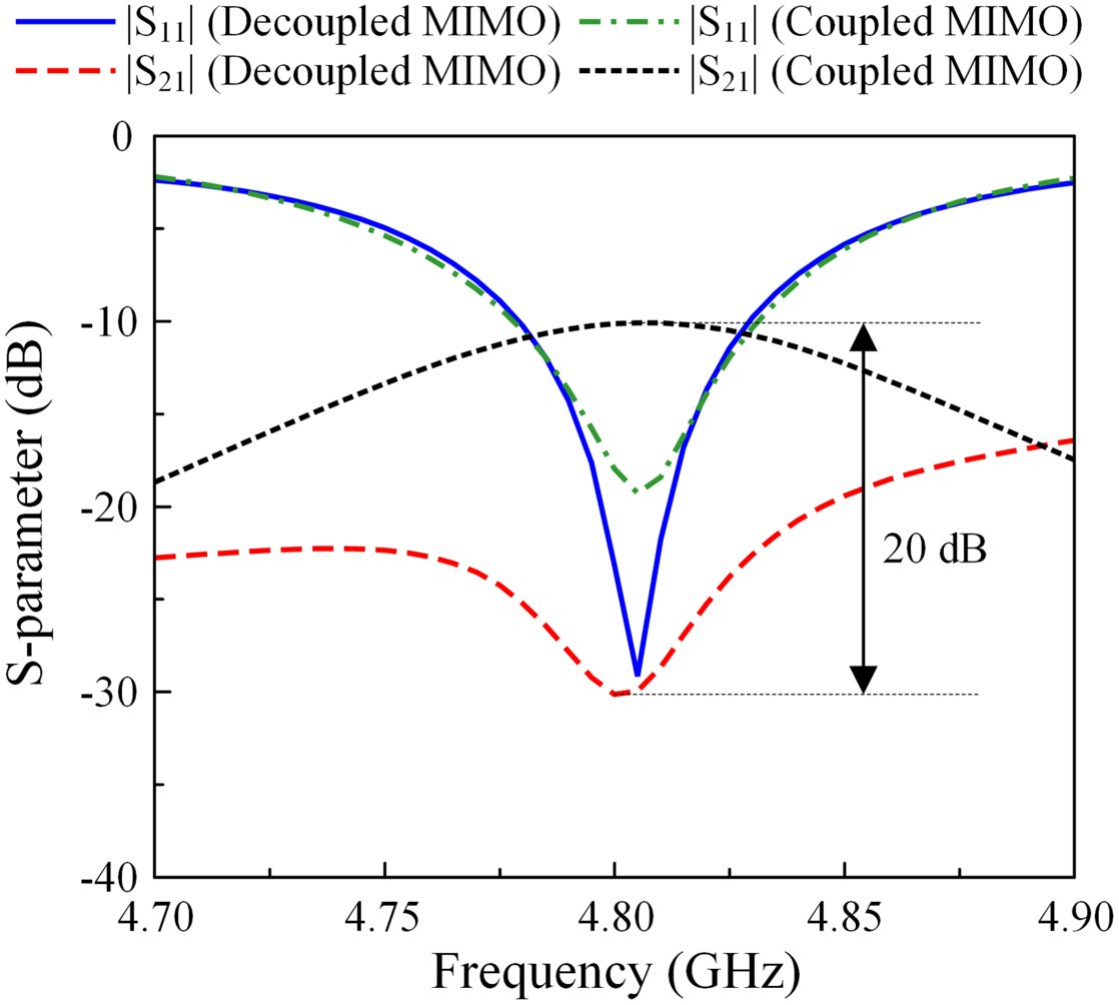
S-parameter of the decoupled and coupled MIMO antennas.

According to this theory, the mutual coupling in the MIMO system can be suppressed if the MIMO elements work in orthogonal modes. Fig 2 shows the simulated E-field distributions on both coupled and decoupled MIMO antennas. Note that these designs are optimized so that the operating frequency is similar at 4.8 GHz. In the coupled MIMO array, the polarizations are similar for both excited and non-excited elements. The operating modes of these patches are TM10. With the presence of the decoupling structure, the operating modes on the MIMO elements are orthogonal. The TM10 mode is excited on the primary radiating patch and *TM*01 is the dominant mode on the non-excited patch. Accordingly, the MIMO array with the shorted stub will perform better isolation than the other. A further demonstration can be observed in Fig 3, which shows the simulated reflection and transmission coefficients of the MIMO arrays with and without decoupling structures. The data indicate that when both MIMO arrays work in a similar band, the isolation values are significantly different. At 4.8 GHz, the isolation of the decoupled MIMO is 30 dB, which is 20 dB larger than the coupled MIMO.

### Optimization process

The optimization process of the proposed 2-element MIMO antenna can be divided into three main steps: operating frequency, impedance matching, and isolation. In general, these performance characteristics can be controlled independently.

**Fig 4:**
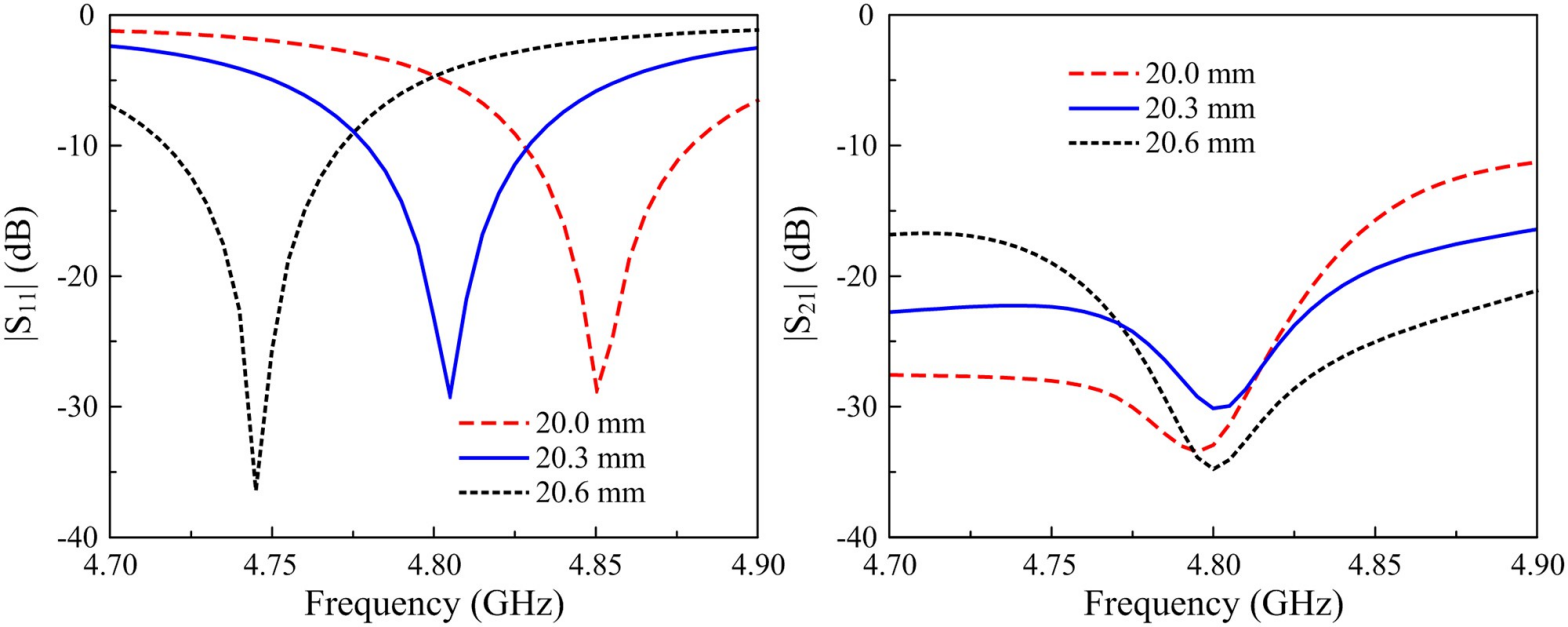
Simulated |*S*_11_| and |*S*_21_| of the 2-element MIMO antenna for different values of *L*_*p*_.

First of all, the resonance frequency of the proposed MIMO antenna can be controlled by tuning the length of the microstrip patch, Lp. Fig 4 shows the antenna’s performances against the variations of Lp. As seen, the S11 resonance increases with the decrease of Lp. Meanwhile, the isolation is almost stable with the variation of this parameter.

**Fig 5:**
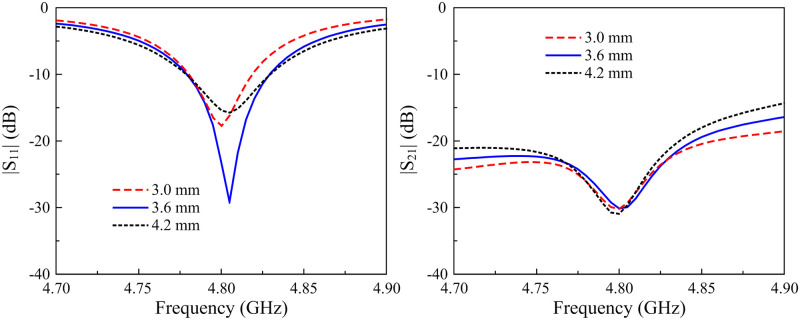
Simulated |*S*_11_| and |*S*_21_| of the 2-element MIMO antenna for different values of *l*_*f*_.

Next, the matching performance is considered. The simulated S-parameter results for different values of lf are illustrated in Fig 5. The data demonstrate that the matching is strongly affected by the feeding position. On the other hand, the effect of lf on the isolation characteristic is insignificant.

**Fig 6:**
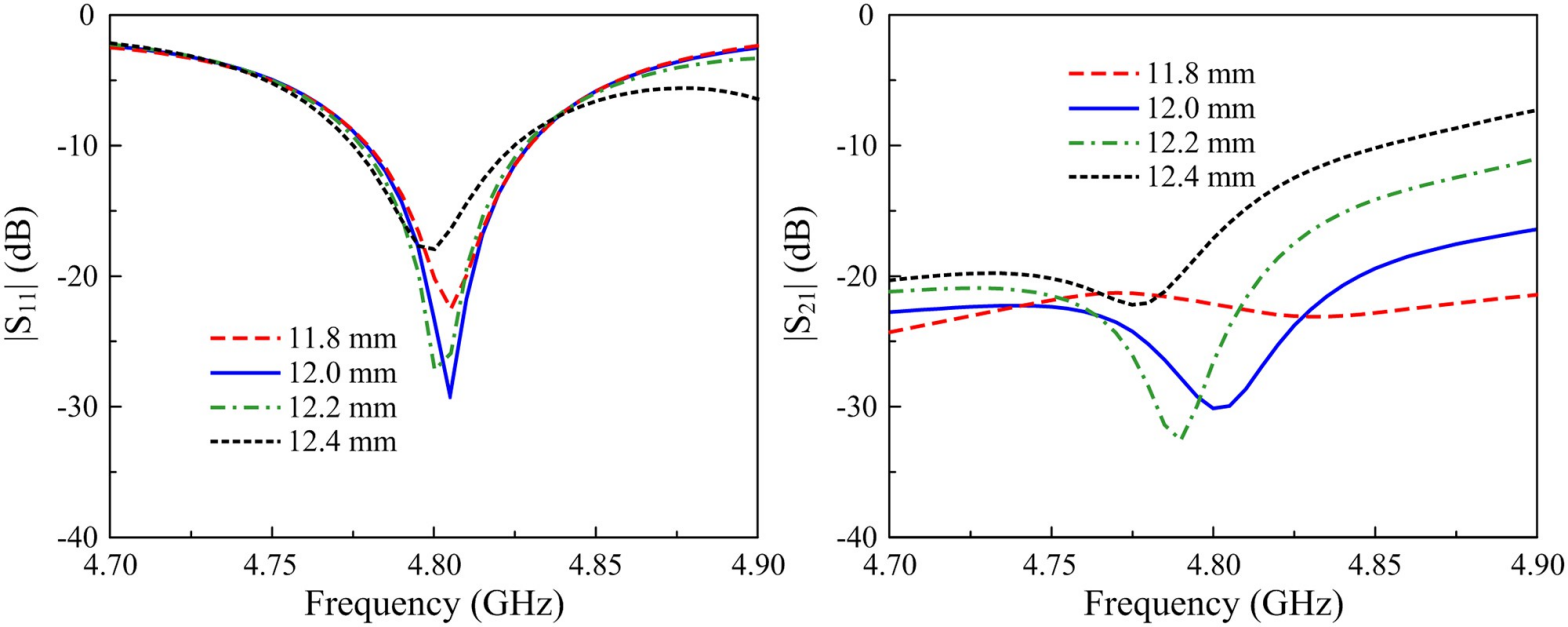
Simulated |*S*_11_| and |*S*_21_| of the 2-element MIMO antenna for different values of *l*.

**Fig 7:**
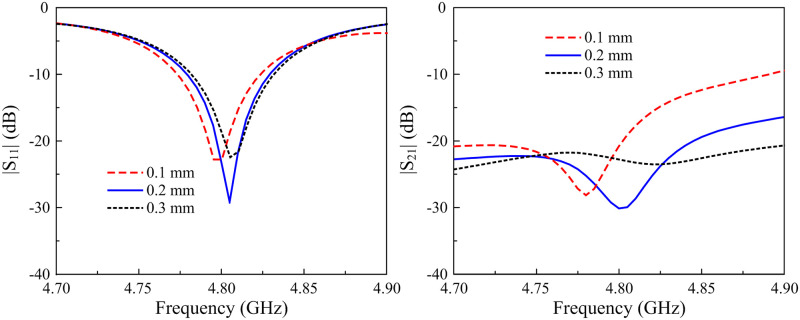
Simulated |*S*_11_| and |*S*_21_| of the 2-element MIMO antenna for different values of *r*_*v*_.

Finally, the isolation optimization is investigated. Figs 6 and 7 show the simulated S-parameter against the variations of *l* and *r*_*v*_. It can be seen that the effect of these parameters on the matching performance is minor. The |*S*_11_| resonance is stable around 4.8 GHz. In contrast, the isolation value is considerably affected. In the |*S*_21_| profile, the lowest |*S*_21_| peak shifts downwards with the increase of *l* and the decrease of *r*_*v*_. With proper values of *l* and *r*_*v*_, the isolation at 4.8 GHz will achieve the best performance.

## Extension to a large-scale array

Various decoupling structures have been reported for patch antenna arrays. However, these proposals are merely capable of alleviating mutual coupling between two adjacent elements. They might not work with multiple-port MIMO systems. In view of this point, a four-element patch array is further designed to demonstrate the decoupling capacity of the proposed decoupling structure in the MIMO patch antenna.

**Fig 8:**
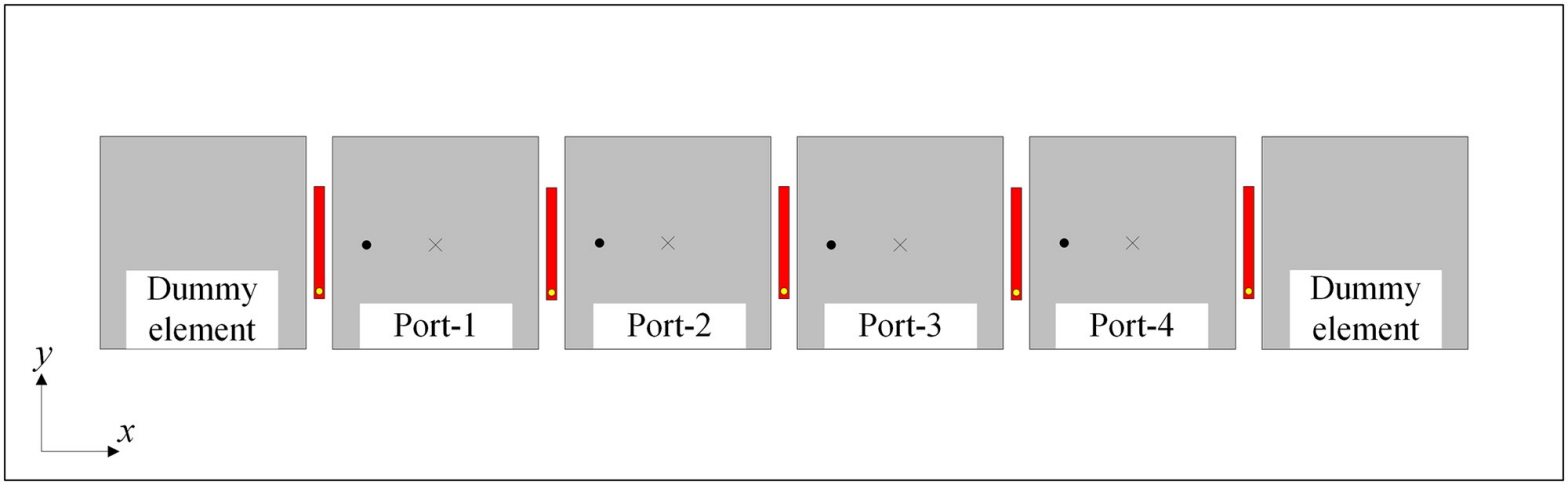
Geometry of the proposed 1 × 4 MIMO antenna.

**Fig 9:**
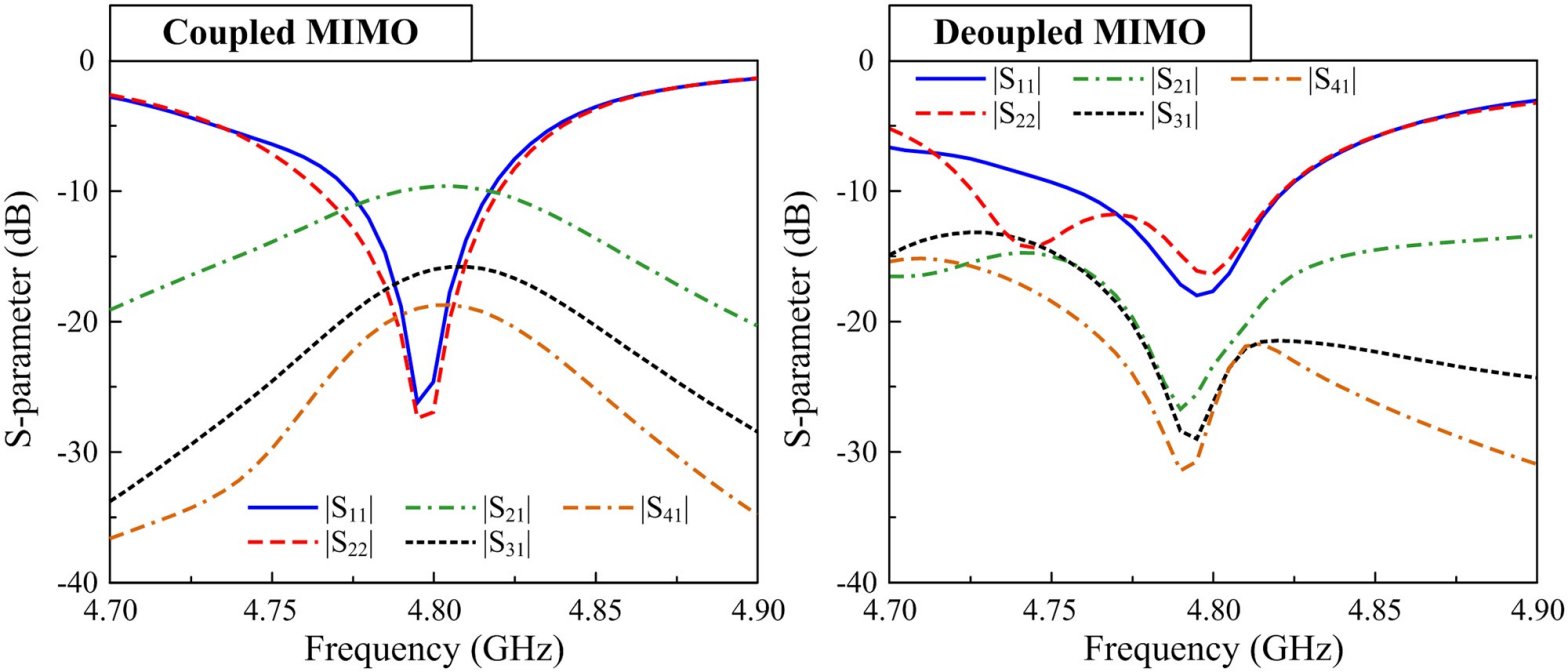
Simulated S-parameter of different 1 × 4 MIMO antennas.

Fig 8 shows the geometry of the proposed 1 × 4 MIMO array. The dimensions of the microstrip patch and the decoupling structure are the same as those of the aforementioned two-element patch MIMO array. Noting that two dummy elements are positioned at both sides of the array to balance the impedance performance for all elements. The simulated S-parameter results with and without decoupling in the four-element patch array are illustrated in Fig 9. The data indicate that in comparison with the coupled MIMO array, the mutual coupling between adjacent and non-adjacent elements is significantly suppressed. Besides, the impedance-matching performances at all feeding ports are good for both coupled and decoupled arrays. Accordingly, the proposed technique is demonstrated as an effective method in mutual coupling reduction for the MIMO patch array with multiple elements. The MIMO array can be extended to a large-scale 1 × N array.

## N-element design procedure

According to the investigation on 2- and 4-element MIMO antenna, the design procedure for an arbitrary N-element MIMO array can be summarized as follows:

- Step 0: Choose the proper substrate materials that are thin and flexible.
- Step 1: Initial setup of the parameters for a 2-element MIMO antenna:
  - Two microstrip patches are arranged in the E-plane coupled configuration;
  - The length of the patch (*L*_*p*_) is chosen about half-effective wavelength at the desired frequency band;
  - The feeding position (*l*_*f*_) is within the patch (*l*_*f*_ < *L*_*p*_/2);
  - The ground stub (*l*, *w*, *r*_*v*_) as the decoupling network is positioned between the MIMO elements;
- Step 2: Tune *l*_*p*_ to achieve the desired operating band (as demonstrated in Fig 4).
- Step 3: Tune *l*_*f*_ to achieve good matching performance (as demonstrated in Fig 5).
- Step 4: Tune *l*, *l*_*v*_ to achieved good isolation (as demonstrated in Figs 6 and 7).
- Step 5: Final tune all design parameters to achieve the best performances in terms of matching and isolation features.
- Step 6: Design an arbitrary N-element MIMO array by increasing the number of MIMO elements and adding two dummy elements.

## Measurement results and discussion

**Fig 10:**
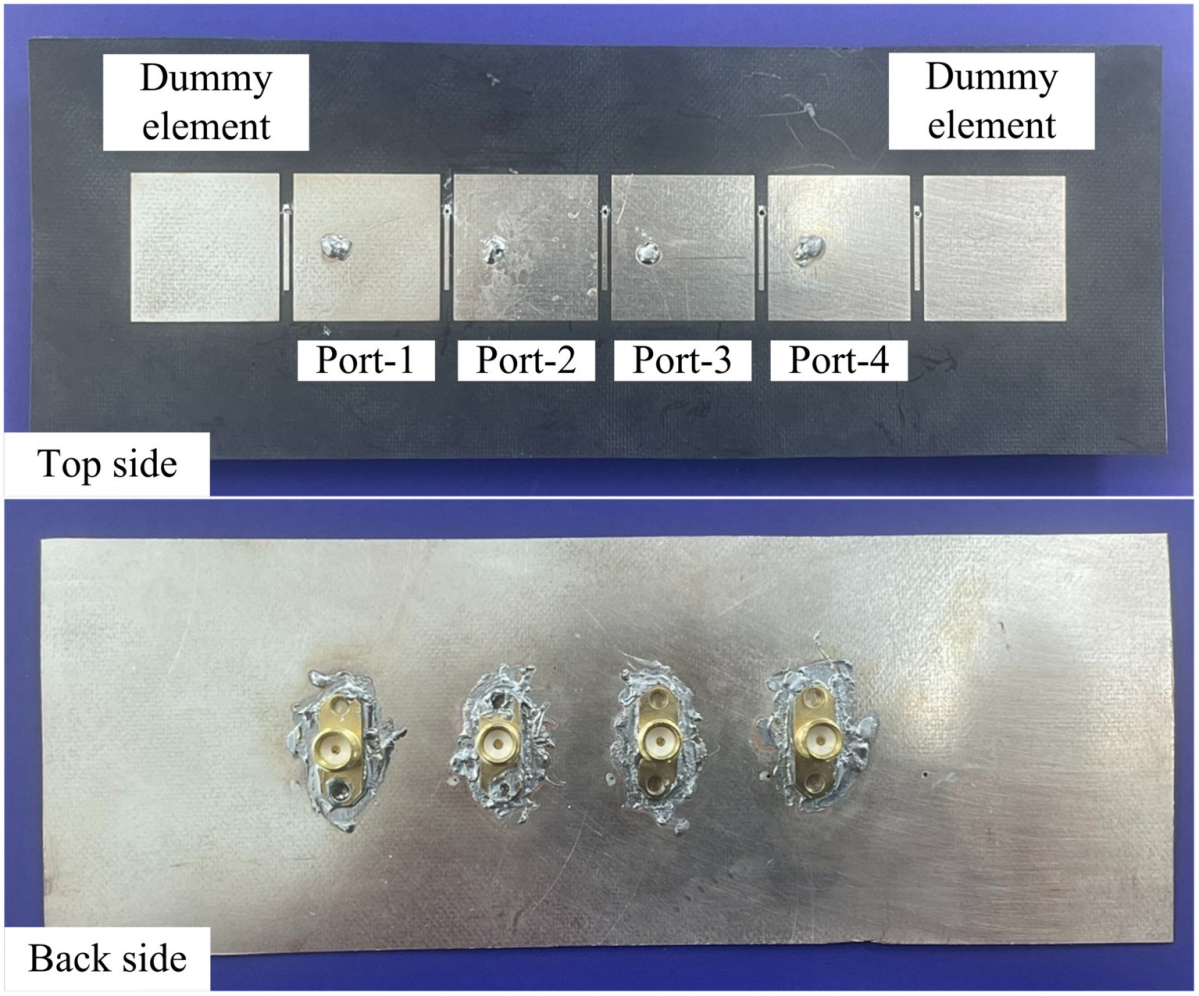
Top view and bottom view of the fabricated 1 × 4 MIMO antenna array.

The 1 × 4 MIMO antenna array is fabricated and then the measurements are implemented on this prototype. Fig 10 shows the photographs of the fabricated MIMO prototype with different views. Furthermore, the MIMO antenna’s far-field characteristics encompassing gain and radiation patterns are evaluated in an anechoic chamber at the Electromagnetic Wave Technology Institute in Seoul, Korea. In the far-field assessment, the MIMO antenna under study operates as the receiver, while a conventional wideband horn antenna is utilized as the transmitter. Overall, there is a strong agreement between the simulations and measurements, with any minor variations attributed to manufacturing tolerances and imperfections in the measurement configuration.

### S-parameter and far-field results

**Fig 11:**
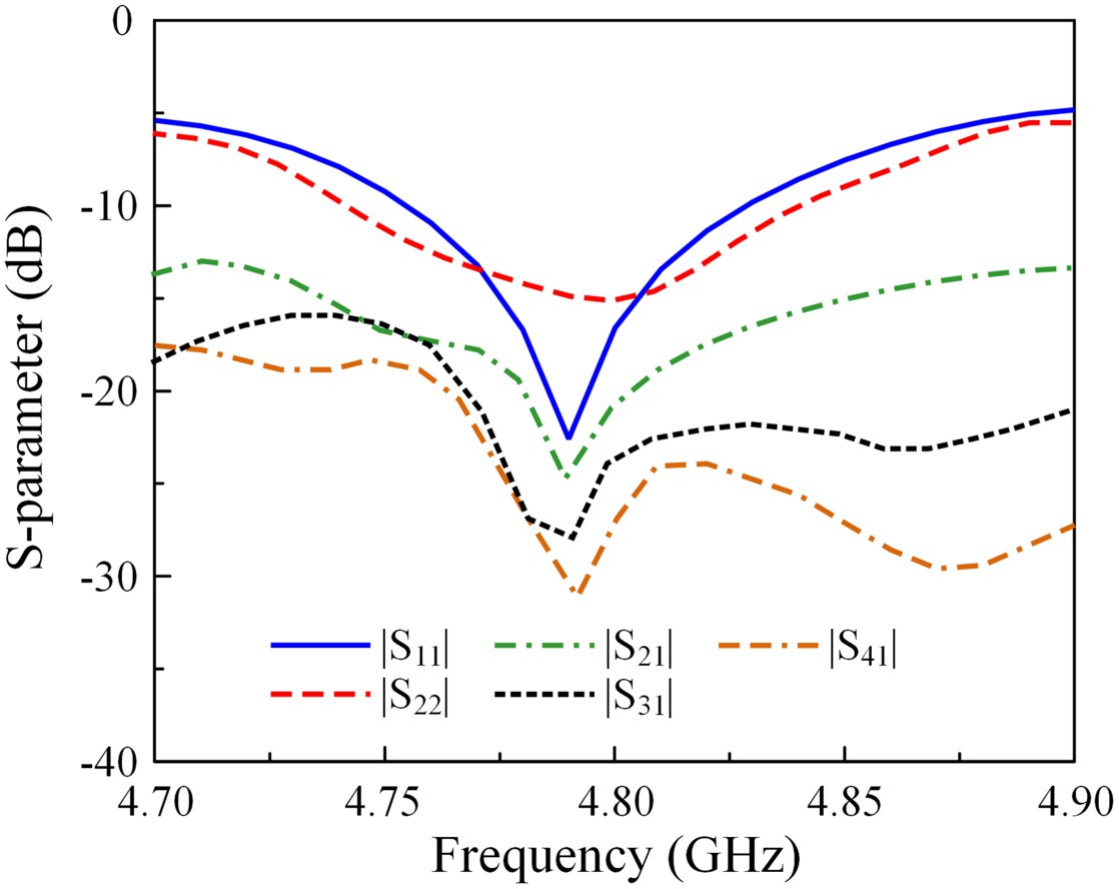
Measured S-parameter of the proposed 1 × 4 MIMO antenna array.

The simulated and measured S-parameters of the fabricated MIMO prototype are illustrated in Fig 11. The measured –10 dB impedance BWs is 1.6% (4.75–4.83 GHz). The measured isolation among the MIMO elements is better than 15 dB across the operating BW. Around 4.8 GHz, the array exhibits the best isolation of better than 20 dB.

**Fig 12:**
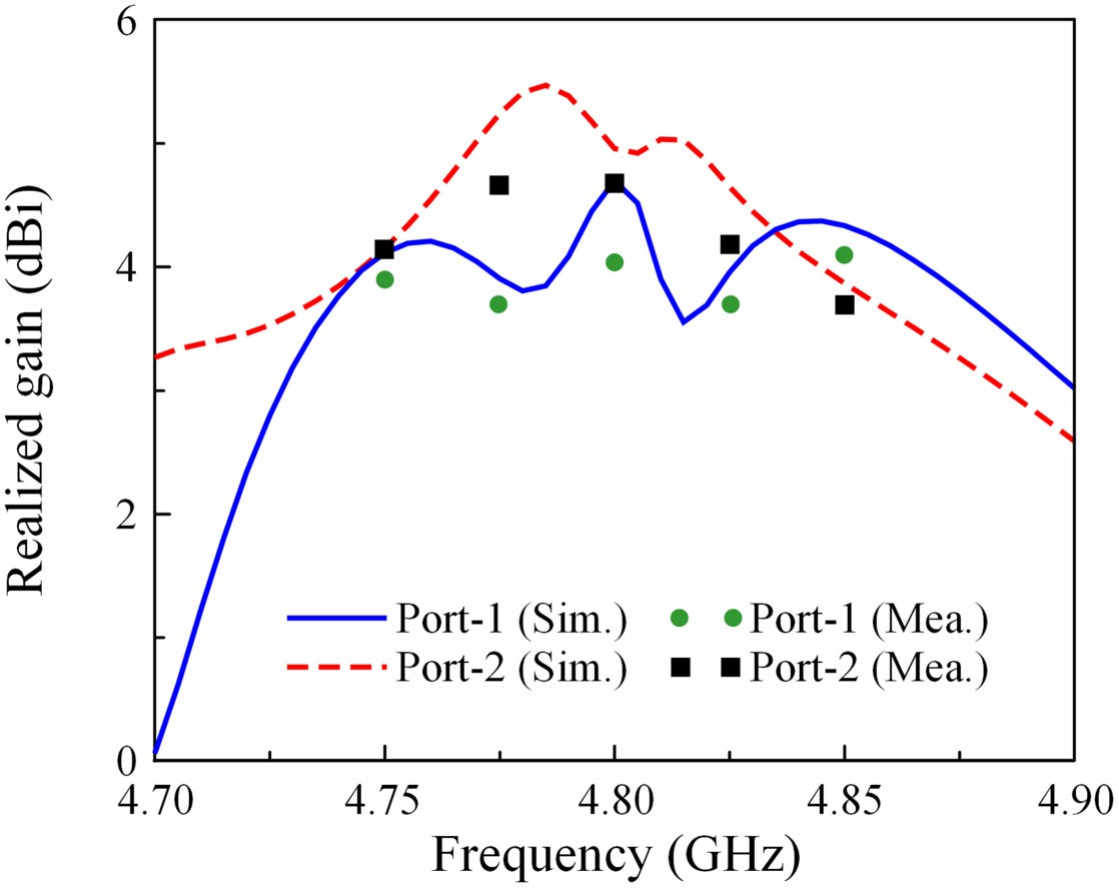
Measured realized gain of the proposed 1 × 4 MIMO antenna array.

**Fig 13:**
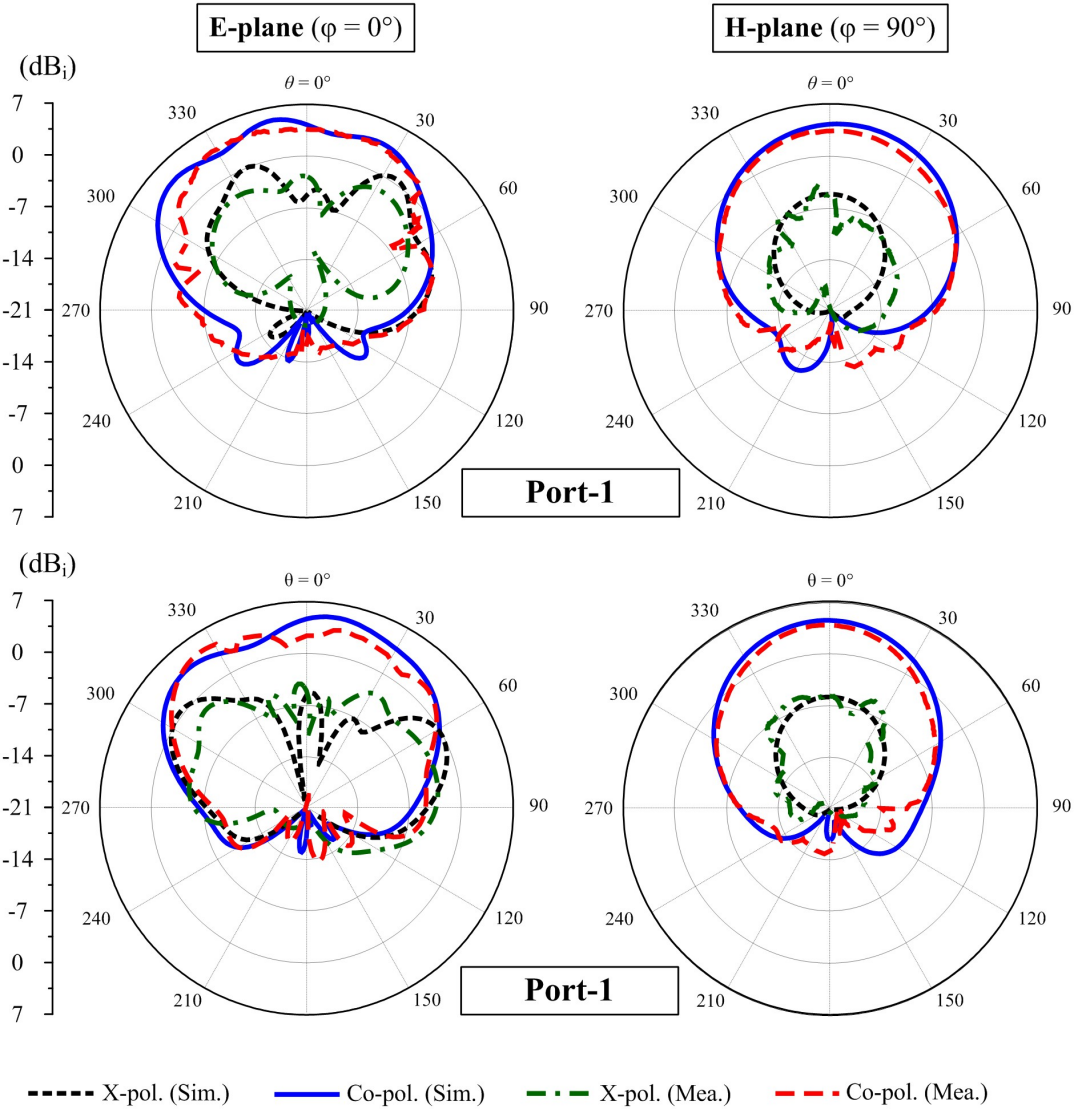
Measured radiation patterns at 4.8 GHz of the proposed 1 × 2 MIMO antenna array with different excitations.

Due to the symmetrical antenna geometry, the far-field performances are only characterized by Port-1 excitation. Fig 12 presents the realized gain results in the broadside direction (+*z*-direction). For the far-field test, when one port is excited, the other is terminated with a 50-*Ω* load. As observed, the simulated and measured gain values are almost similar. The antenna has a maximum gain of 4.7 dBi within the operating band from 4.75 to 4.83 GHz. The radiation patterns at 4.8 GHz of the proposed antenna are plotted in Fig 13. It can be seen that in both E- and H-plane, the proposed antenna performs good bore-sight radiation. The radiation patterns in H-plane are quite symmetric in the broadside direction. On the other hand, due to the effect of the other elements in the E-plane, the radiation patterns are slightly distorted in several directions, but they are acceptable.

### MIMO performances

The MIMO performances of the proposed MIMO array are evaluated through several important parameters including the envelope correlation coefficient (ECC) and the diversity gain (DG). The ECC and DG are calculated based on the S-parameter Eqs 1, 2 and 3.

$$\begin{array}{c} ECC_{ij}=\frac{{\left|R_{ii}^{*}*T_{ij}+T_{ji}^{*}*S_{jj}\right|}^{2}}{\left(1-{\left|R_{ii}\right|}^{2}-{\left|T_{ji}\right|}^{2})(1-{\left|R_{jj}\right|}^{2}-{\left|T_{ij}\right|}^{2}\right)} \end{array}$$ (1)

$$\begin{array}{c} \rho_{eij}=\frac{{\left|{\;\int \int \;}_{0}^{4\pi }[\vec{R_{i}}\left(\theta ,\varphi \right)\times \vec{R_{j}}\left(\theta ,\varphi \right)]d\Omega \right|}^{2}}{{\;\int \int \;}_{0}^{4\pi }{\left|\vec{R_{i}}\left(\theta ,\varphi \right)\right|}^{2}d\Omega {\;\int \int \;}_{0}^{4\pi }{\left|\vec{R_{j}}\left(\theta ,\varphi \right)\right|}^{2}d\Omega } \end{array}$$ (2)

$$\begin{array}{c} D_{gain}=10\sqrt{1-{\left|ECC_{ij}\right|}^{2}} \end{array}$$ (3)

**Fig 14:**
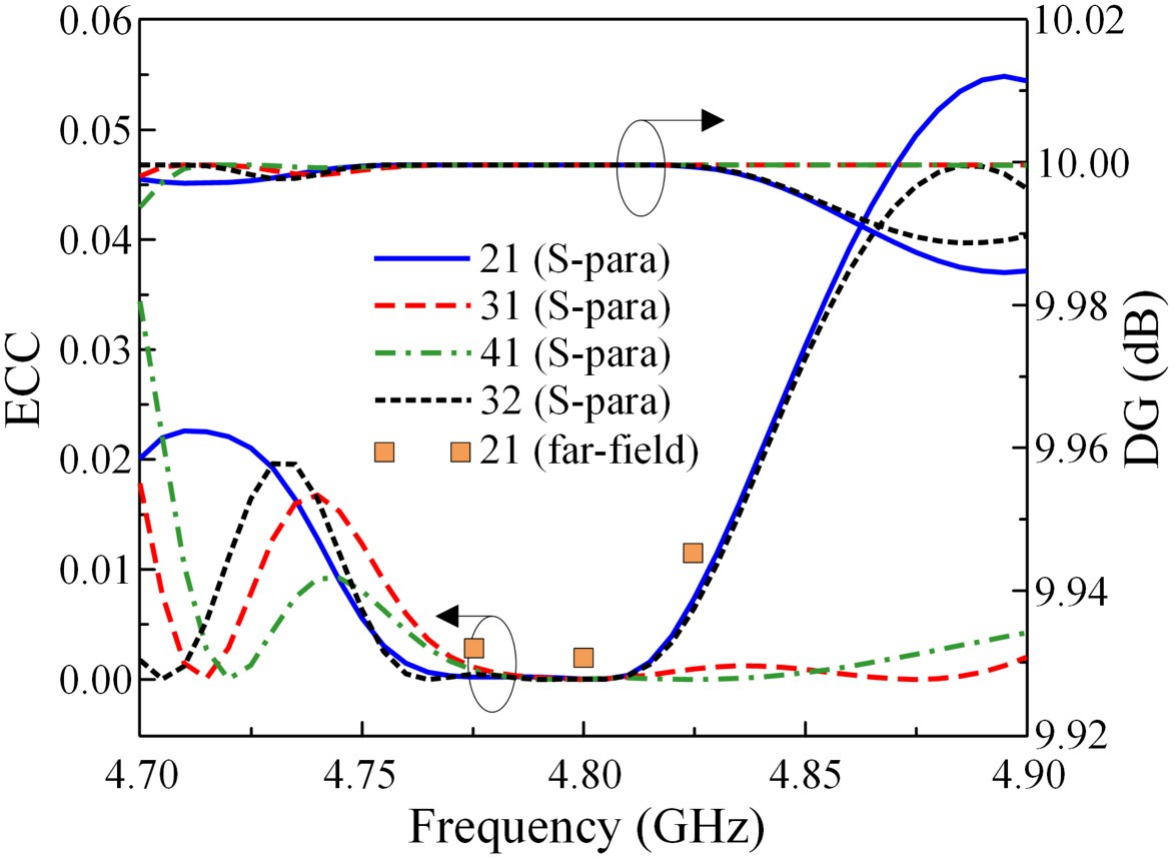
Calculated ECC and DG of the proposed 1 × 4 MIMO antenna array.

Here, *i*, *j* are port numbers, and *R*, *T* are the reflection and transmission coefficients. *Ω* is the solid angle of the far-field radiation patterns $\vec{R_{i}}\left(\theta ,\varphi \right)$ and $\vec{R_{j}}\left(\theta ,\varphi \right)$. The calculated ECC and DG results are illustrated in Fig 14. It can be seen that the observed ECC is significantly below the allowable threshold of 0.5 throughout the operating frequency range. The DG is nearly at its theoretical maximum value of approximately 10.

### Comparison with related works

The comprehensive comparison among microstrip patch MIMO antenna arrays is summarized and given in Table 1. Note that various flexible MIMO antennas using monopole structures have been published in the literature. However, the operating principle and radiation features are completely different from the microstrip patch antennas. Therefore, the comparison with the flexible monopole MIMO is not appropriate and not included in Table 1. As observed, although wideband operation and high isolation can be achieved in designs [7, 11, 15–18], these MIMO arrays cannot work in flexible mode, which reduces the integration capability of such designs. Besides, the edge-to-edge element spacings are always higher than 0.03λ. In comparison with flexible MIMO arrays [24, 25], the proposed antenna has the advantage of smaller element spacing and the capability to extend to a large-scale 1 × N MIMO array.

**Table 1:** Comparison among microstrip patch MIMO antennas.

Ref.	Profile	Edge spacing (λ_0_)	BW (%)	Isolation (dB)	Extensibility	Flexibility
[7]	0.09	0.05	14.8	>20	No	No
[11]	0.02	0.03	<3.0	>20	No	No
[15]	0.05	0.04	6	>20	Yes	No
[16]	0.03	0.07	<3.0	>20	Yes	No
[17]	0.04	0.18	3.4	>20	Yes	No
[18]	0.04	0.14	2.9	>20	Yes	No
[24]	0.03	0.08	<3.0	>20	No	Yes
[25]	0.02	0.09	27.6	>34	No	Yes
Prop.	0.01	0.03	<3.0	>20	Yes	Yes

## Conformal test

**Fig 15:**
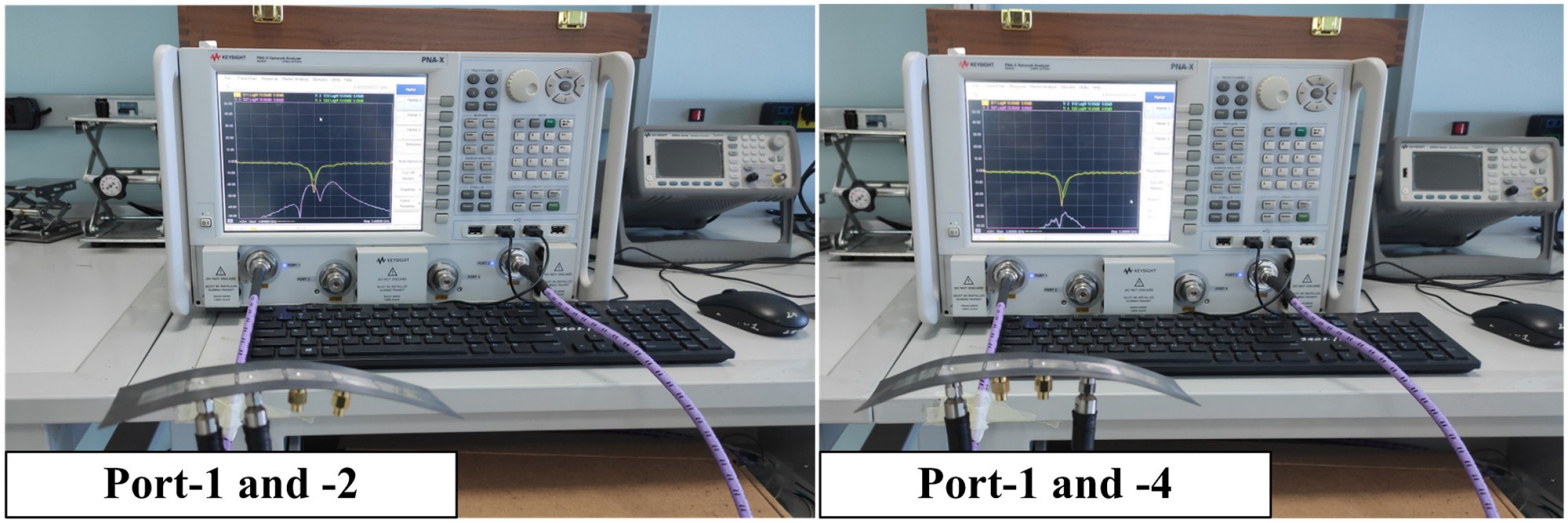
Measurement of the proposed 1 × 4 MIMO antenna array in bending mode.

The proposed MIMO antenna is modeled on the thin substrate, the proposed antennas are expected to work effectively when mounting on curved surfaces. For measurement shown in Fig 15, the antenna is gradually bent along x-axis until it reaches a radius of 80 mm.

**Fig 16:**
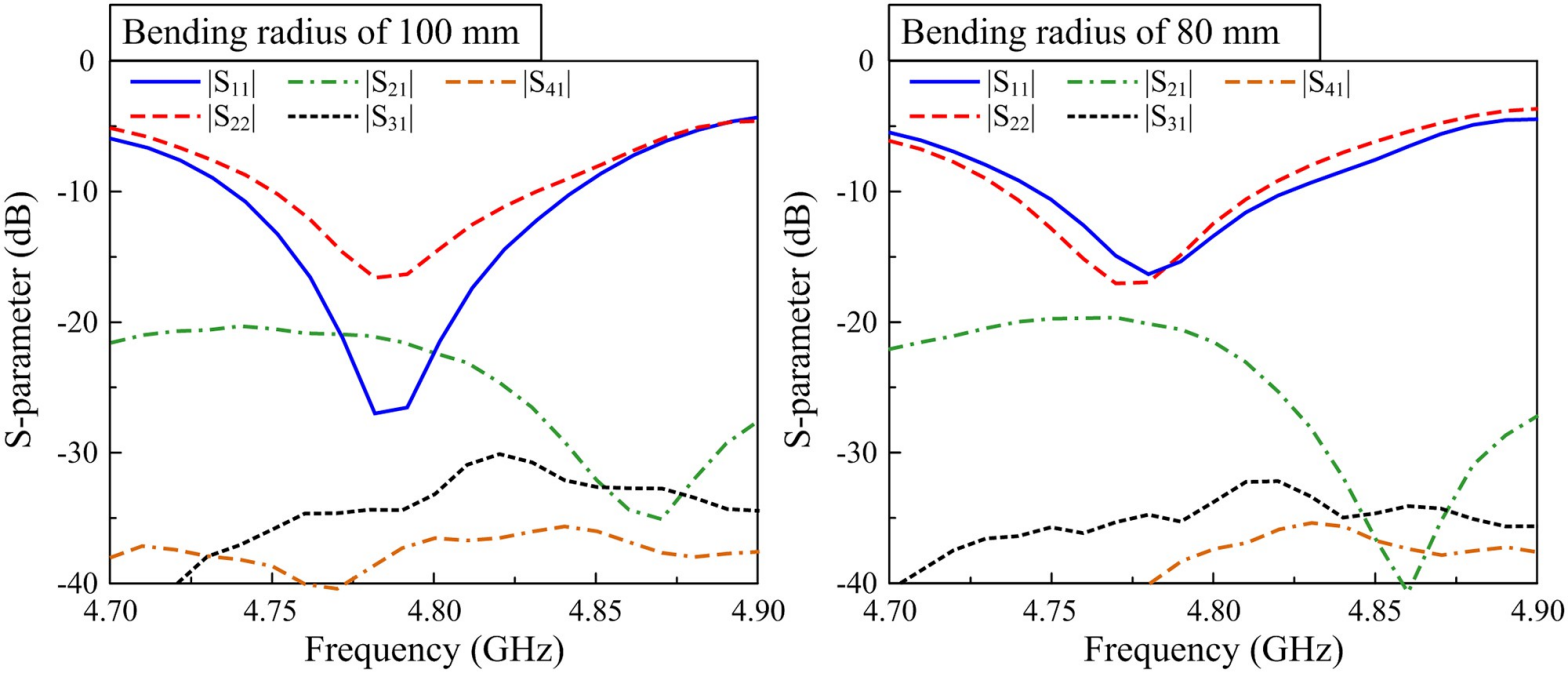
Measurement S-parameter of the proposed 1 × 4 MIMO antenna array with different bending radius.

The measured S-parameter results of the fabricated MIMO array in bending conditions are presented in Fig 16. Here, two different bending radii of 100 mm and 80 mm are measured. The measured data indicates that when the antenna is bent, the measured reflection coefficients are stable at around 4.8 GHz. Regarding the isolation, it can be seen that the isolations among the MIMO elements in both cases are lower than 20 dB. This demonstrates the efficiency of the proposed MIMO array when working in flexible mode.

## Conclusion

This paper presents a flexible MIMO antenna array with multiple ports. An array of microstrip patch antennas are arranged in the E-plane configuration with edge-to-edge element spacing of 0.032λ. In order to suppress the mutual coupling, shorted quarter-wavelength stubs are positioned between the MIMO elements. A 1 × 4 MIMO array prototype is fabricated and tested to validate the feasibility of the proposed concept. The measured operating BW of about 1.6% with isolation of greater than 15 dB. At the center frequency of 4.8 GHz, the isolations among MIMO elements are always better than 20 dB. The MIMO array has also been demonstrated to work effectively in the bending mode. The proposed antenna can be a potential candidate in various modern electronic devices working in the 4.8 GHz N79-5G frequency band.
